# Regulation of hypothalamic neuropeptides gene expression in diet induced obesity resistant rats: possible targets for obesity prediction?

**DOI:** 10.3389/fnins.2015.00187

**Published:** 2015-06-08

**Authors:** Carlo Cifani, Maria V. Micioni Di Bonaventura, Mariangela Pucci, Maria E. Giusepponi, Adele Romano, Andrea Di Francesco, Mauro Maccarrone, Claudio D'Addario

**Affiliations:** ^1^Pharmacology Unit, School of Pharmacy, University of CamerinoCamerino, Italy; ^2^Intramural Research Program, National Institute on Drug Abuse/National Institutes of HealthBaltimore, MD, USA; ^3^Faculty of Bioscience and Technology for Food, Agriculture and Environment, University of TeramoTeramo, Italy; ^4^Department of Physiology and Pharmacology “V. Erspamer,” Sapienza University of RomeRome, Italy; ^5^Center of Integrated Research, Campus Bio-Medico University of RomeRome, Italy; ^6^European Center for Brain Research (CERC)/Santa Lucia FoundationRome, Italy

**Keywords:** diet induced obesity, hypothalamus, neuropeptides, gene expression, DNA methylation, high fat diet

## Abstract

Several factors play a role in obesity (i.e., behavior, environment, and genetics) and epigenetic regulation of gene expression has emerged as a potential contributor in the susceptibility and development of obesity. To investigate the individual sensitivity to weight gain/resistance, we here studied gene transcription regulation of several hypothalamic neuropeptides involved in the control of energy balance in rats developing obesity (diet-induced obesity, DIO) or not (diet resistant, DR), when fed with a high fat diet. Rats have been followed up to 21 weeks of high fat diet exposure. After 5 weeks high fat diet exposure, the obese phenotype was developed and we observed a selective down-regulation of the orexigenic neuropeptide Y (NPY) and peroxisome proliferator-activated receptor gamma (PPAR-γ) genes. No changes were observed in the expression of the agouti-related protein (AgRP), as well as for all the anorexigenic genes under study. After long-term high fat diet exposure (21 weeks), NPY and PPAR-γ, as well as most of the genes under study, resulted not be different between DIO and DR, whereas a lower expression of the anorexigenic pro-opio-melanocortin (POMC) gene was observed in DIO rats when compared to DR rats. Moreover we observed that changes in NPY and POMC mRNA were inversely correlated with gene promoters DNA methylation. Our findings suggest that selective alterations in hypothalamic peptide genes regulation could contribute to the development of overweight in rats and that environmental factor, as in this animal model, might be partially responsible of these changes via epigenetic mechanism.

## Introduction

Obesity is the consequence of an imbalance between energy intake and expenditure. The prevalence of obesity all over the world suggests that there is a fundamental weakness in the regulation of appetite and energy homeostasis. Feeding behavior and body weight are controlled through complex interactions between the central nervous system (CNS) and peripheral organs. Short- and long-term hormonal and neural signals regulate food intake and energy expenditure through the modulation of orexigenic and anorexigenic neuropeptides expression in the hypothalamus and in other brain regions involved in the control of the homeostasis (Woods et al., 1998; Schwartz et al., 2000). Critical elements of this control system are hormones secreted in proportion to body adiposity, including leptin and insulin, and the CNS targets upon which they act (Hewson et al., 2002).

In recent years, several evidences have been accumulated on the effects of a large number of hypothalamic neuropeptides on food intake, including neuropeptide Y (NPY), galanin, cocaine and amphetamine regulated transcript (CART), melanin concentrating hormone (MCH), agouti-related protein (AgRP), and pro-opio-melanocortin (POMC)-derived peptides, such as α-melanocyte-stimulating hormone (α-MSH) and β-endorphin (Woods et al., 1998). The activation of NPY/AGRP neurons has an orexigenic effect, promoting food intake, whereas the activation of POMC/CART neurons evoke an anorexigenic effect (Barsh and Schwartz, 2002).

These two sets of neurons receive inputs from several endocrine hormones such as leptin, secreted from the adipose tissue, which exerts its effects via the leptin receptor (lepR). In particular leptin regulates body weight homeostasis and energy balance (Friedman and Halaas, 1998; Meister, 2000) by acting at hypothalamic level inhibiting the NPY/AGRP pathway and stimulating POMC/CART neurons (Bell et al., 2005).

Recently a crucial role has been also proposed for the peroxisome proliferator-activated receptor gamma (PPAR-γ), already known to be relevant for energy homeostasis in POMC hypothalamic neurons (Long et al., 2014). In particular Long and his group demonstrated that PPAR-γ mediates several cellular, biological, and functional adaptations of POMC neurons in mice exposed to a fat enrich diet (Long et al., 2014).

Diet-induced obesity in rats provides a useful animal model sharing several common features with human obesity (Levin and Dunn-Meynell, 2000), including a polygenic mode in inheritance. Many studies have shown in different strains of rodents that, after exposure to a high fat diet, some animals become obese (DIO) while others remain lean (DR) (Surwit et al., 1997; Bergen et al., 1999; Levin and Dunn-Meynell, 2000; Tian et al., 2004). These weight-gain patterns appear to be inherited as polygenic (Levin et al., 1997, 2003; Levin and Govek, 1998). Before the onset of obesity, outbred DIO-prone rats show a number of abnormalities of nervous system function, which might predispose them to develop obesity when offered a 31% fat, high-energy diet (Levin and Routh, 1996). The molecular mechanisms of this different response to dietary factors are still largely unknown.

Obesity is a classic example of interaction between environment (e.g., diet) and heredity and among the several mechanisms that could lead to interindividual differences to develop obesity, the epigenetic regulation of gene expression has emerged in the last years as a potentially important contributor (Lavebratt et al., 2012). Epigenetic processes consist of mitotically heritable, but reversible, changes in gene accessibility that occur without a change in the genomic DNA sequence (Russo et al., 1996). The main epigenetic mechanisms essentially include DNA methylation and histone modifications (Feng et al., 2007). DNA methylation, in particular, consists of the transfer of a methyl group to position 5 of the cytosine pyrimidine ring of a cytosine guanine dinucleotide (CpG), which ultimately blocks the binding of transcription factors causing chromatin compaction and gene silencing. Reports have identified epigenetic modifications in the CNS in response to altered diet, particularly in the prenatal or early postnatal time period (Vucetic et al., 2010a,b). However, few studies investigated the postnatal period and, in this frame, the search for gene promoters susceptible to epigenetic regulation, with a role in the development of obesity, could be of great interest.

On the basis of above considerations, in the present study we investigated the individual sensitivity to weight gain/resistance, following a hypercaloric diet. In particular we evaluated the hypothalamic gene expression and possible epigenetic regulation of selected neuropeptides involved in the control of energy balance in rats that develop obesity (diet-induced obesity, DIO), or do not develop obesity (diet resistant, DR), when fed with a high fat diet. Namely, we studied the transcriptional regulation of the peptides NPY, AGRP, POMC, CART as well as of the two receptors PPAR-γ and lepR.

## Methods

### Subjects and diet composition

Male Sprague Dawley rats (Charles River; total *n* = 56; 175–200 g at the beginning of the experiments) were used. Rats were housed in individual cages under 12 h:12 h light/dark cycle (lights on at 9:00 a.m.) with access to food and water *ad libitum* for 2 weeks before the experiments. They were kept in a room at constant temperature (20–22°C) and humidity (45–55%). All experiments were performed in accordance with the European directive 86/609/EEC governing animal welfare and protection, which is acknowledged by Italian Legislative Decree n. 116, January 27, 1992. Rats were randomly divided into two groups with comparable mean body weight (no significant difference). The first group (*n* = 6) was the control group and was fed with standard laboratory chow *ad libitum* (4RF18, Mucedola, Settimo Milanese, Italy; 2.6 kcal/g); the second group (*n* = 50), was fed with high energy diet (45% fat, 35% Carbohydrate, 20% Protein) *ad libitum* (D12451, Research Diets, Inc., New Brunswick, NJ; 5.24 kcal/g).

After 5 weeks, rats fed with high fat diet showed a different sensitivity to the diet so that they were divided in two different group: the rats which increased significantly their body weight in comparison to the control group were designated as diet induced obesity (DIO) rats (*n* = 39), while the rats that gain the same body weight of control group were designated as diet resistance (DR) rats (*n* = 11). At the end of the 5 weeks, 7 DIO rats and 5 DR were sacrificed by decapitation. Brains were quickly removed, placed in an ice-cold matrix and hypothalamus was collected. The remaining rats, 32 DIO and 6 DR rats were maintained on their respective diets for 21 weeks. At the end of the 21st week, 6 DIO and 6 DR were sacrificed by decapitation and brains were collected to be analyzed. Body weight and food intake between sacrificed rats (6) and remained rats (26) were not significantly different (*P* > 0.05).

Body weight and food intake were daily measured.

### Real-time qPCR (RT-qPCR)

Total RNA was isolated from the hypothalami, according to the method of Chomczynski and Sacchi (2006). RT-PCR reactions were performed using the RevertAid H Minus First Strand cDNA Synthesis Kit (Thermo Scientific, Waltham, MA, USA). The relative abundance of each mRNA species was assessed by quantitative real-time RTPCR (qRTPCR), using SensiFAST SYBR No-ROX Kit (Bioline) on a DNA Engine Opticon 2 Continuous Fluorescence Detection System (MJ Research, Waltham, MA, USA). All data were normalized to the endogenous reference genes β-actin and GAPDH. The primers used for PCR amplification are provided in Table 1S.

One μl of the first strand cDNA product was used for amplification in triplicate in 20 μl reaction solution, containing 10 μl of SensiFAST SYBR No-ROX Kit and 10 pmol of each primer. The following PCR program was used: 95°C for 10 min, followed by 50 amplification cycles of 95°C for 10 s, 57°C for 30 s, and 72 for 30 s. Relative expression of different gene transcripts was calculated by the Delta-Delta Ct (DDCt) method and converted to relative expression ratio (2^−DDCt^) for statistical analysis (Livak and Schmittgen, 2001). All data were normalized to the endogenous reference genes glyceraldehyde-3-phosphate dehydrogenase and beta-actin expression, properly validated to confirm that their expression was unaffected by our experimental condition.

### Analysis of DNA methylation

Methylation status of gene promoter regions was determined using pyrosequencing of bisulfite converted DNA. Details of pyrosequencing assays used including primer sequences and QIAGEN (Hilden, Germany) assay names are provided in Table 1S. After DNA extraction, 0.5 μg of DNA from each sample was treated with bisulfite, using a DNA methylation kit (Zymo Research, Orange, CA, USA). Bisulfite treated DNA was amplified by PyroMark PCR Kit (Qiagen) according to manufacturer's protocol. PCR conditions were as follows: 95°C for 15 min, followed by 45 cycles of 94°C for 30 s, 56°C for 30 s, 72°C for 30 s, and finally, 72°C for 10 min. PCR products were verified by agarose electrophoresis. Pyrosequencing methylation analysis was conducted using the PyroMark Q24 (Qiagen). The level of methylation was analyzed using PyroMark Q24 Software (Qiagen), which calculates the methylation percentage [mC/(mC + C)] for each CpG site, allowing quantitative comparisons (mC is methylated cytosine, C is unmethylated cytosine).

### Statistical analysis

In behavioral experiments, data were analyzed by Two-Way ANOVA with the animal group as the between-subject variable and time as the within-subject variable, followed by *post-hoc* comparison carried out by the Bonferroni test.

In molecular biology studies, data were analyzed by One-Way ANOVA or Two-Way ANOVA with the animal group as the between-subject variable and CpG sites as the within-subject variable, followed by *post-hoc* comparison carried out by the Bonferroni test. Statistical significance was set at *P* < 0.05.

## Results

### High fat diet effects on body weight and food intake

At the beginning of the study, body weight of rats in the high fat diet group (265.4 ± 2.4 g) did not differ significantly from that of the rats in the control group (267 ± 6.5 g) [*F*_(1, 54)_ = 0.05; *P* > 0.05]. At the end of the fifth week, Two-Way ANOVA showed a significant difference in body weight among the groups (Groups: [*F*_(2, 53)_ = 5.81; *P* < 0.01]; Time [*F*_(4, 212)_ = 566.63; *P* < 0.01]; Interaction: [*F*_(8, 212)_ = 5.94; *P* < 0.01]). At the fifth week time point, *post-hoc* test showed that the average body weight of DIO rats (457.3 ± 4.0 g) began to be significantly higher compared to DR rats (410, 1 ± 7.4 g) (*P* < 0.05) and control group (429.8 ± 6.8 g) (*P* < 0.05).

**Figure 1:**
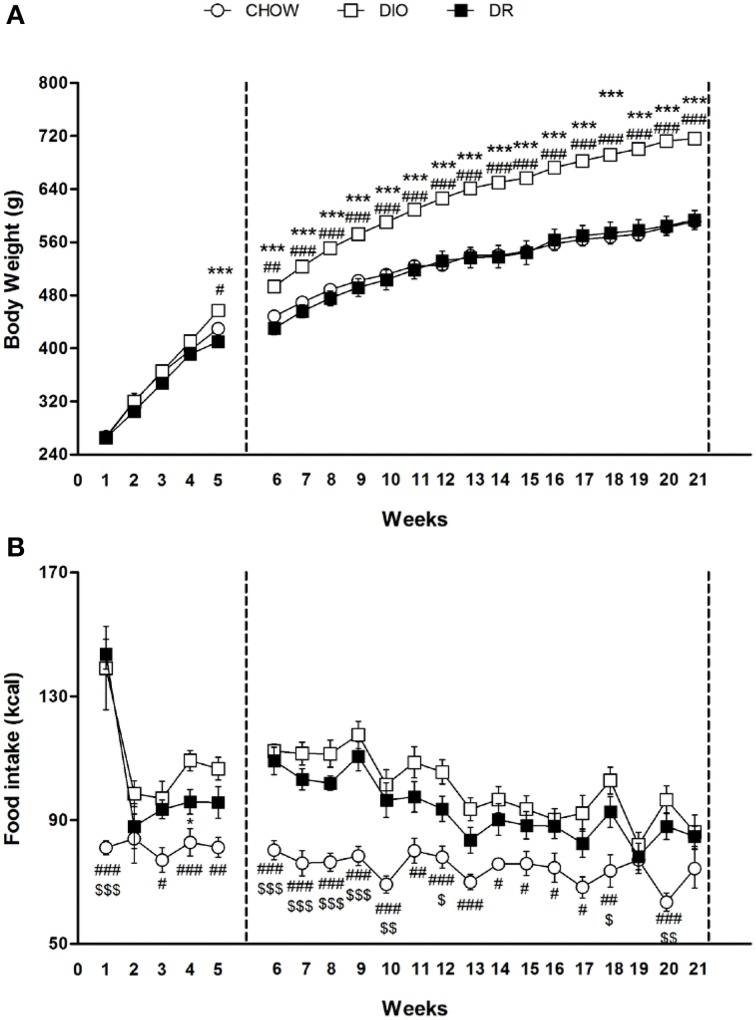
**(A)** Body weight measured weekly for chow, DIO and DR rats. ^#^*P* < 0.05, ^##^*P* < 0.01, ^###^*P* < 0.001 DIO vs. chow; ^***^P < 0.001 DIO vs. DR. **(B)** Cumulative food intake measured weekly for chow, DIO and DR rats. ^#^*P* < 0.05, ^##^*P* < 0.01, ^###^*P* < 0.001 chow vs. DIO; ^$^*P* < 0.05, ^$$^ *P* < 0.01, ^$$$^*P* < 0.001 chow vs. DR; ^*^P < 0.05 DR vs. DIO.

At the end of the 21st week, Two-Way ANOVA showed a significant difference in body weight among the groups (Groups: [*F*_(2, 41)_ = 48.30; *P* < 0.01]; Time [*F*_(15, 615)_ = 327.86; *P* < 0.01]; Interaction: [*F*_(30, 615)_ = 12.93; *P* < 0.01]). At the last time point (21st week) of free access to high fat diet, *post-hoc* test showed that body weight of DIO rats was significantly higher (716.4 ± 8.4 g) in comparison to body weight of rats fed with standard diet (591.0 ± 2.2 g) (*P* < 0.05). Body weight of DR animals (593.5 ± 14.4 g) fed with high fat diet was comparable to body weight of rats fed with standard diet (*P* > 0.05). These rats were resistant to develop obesity (Figure 1A).

Overall ANOVA showed a significant different in energy intake (kcal) among the three groups in the first 5 weeks (Groups: [*F*_(2, 53)_ = 20.39; *P* < 0.01]; Time [*F*_(4, 212)_ = 14.85; *P* < 0.01]; Interaction: [*F*_(8, 212)_ = 3.74; *P* < 0.01]) and from week 6 to week 21 (Groups: [*F*_(2, 41)_ = 18.52; *P* < 0.01]; Time [*F*_(15, 615)_ = 13.85.; *P* < 0.01]; Interaction: [*F*_(30, 615)_ = 2.18; *P* < 0.01]). Significant differences at each time point are expressed in the Figure 1B.

### Regulation of genes transcription

**Figure 2:**
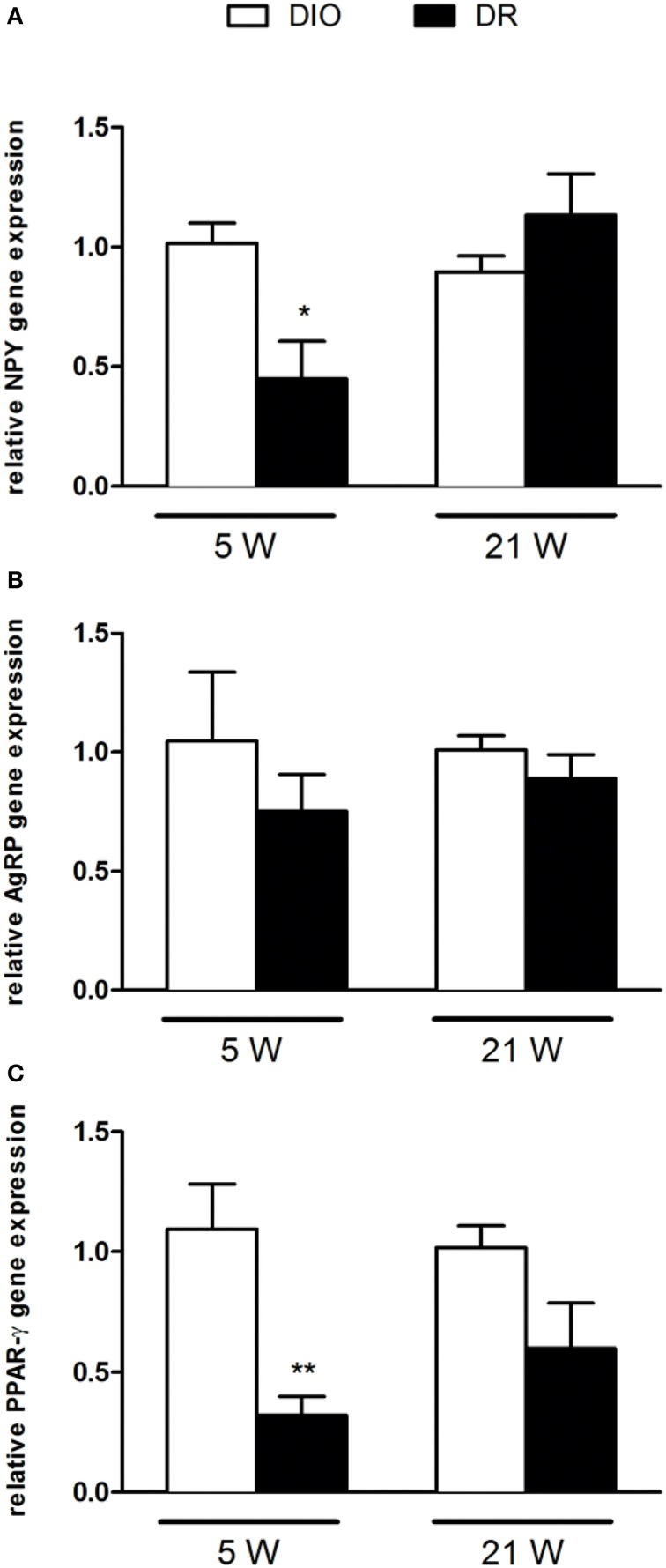
**Gene expression analysis of (A) NPY, (B) AgRP, and (C) PPAR-γ after 5 and 21 weeks of high fat diet exposure**. Gene expression was normalized to glyceraldehyde-3-phosphate dehydrogenase and β-actin. Values are expressed as mean ± SEM of 6–8 rats. ^*^P < 0.05, ^**^P < 0.01 vs. DIO.

**Figure 3:**
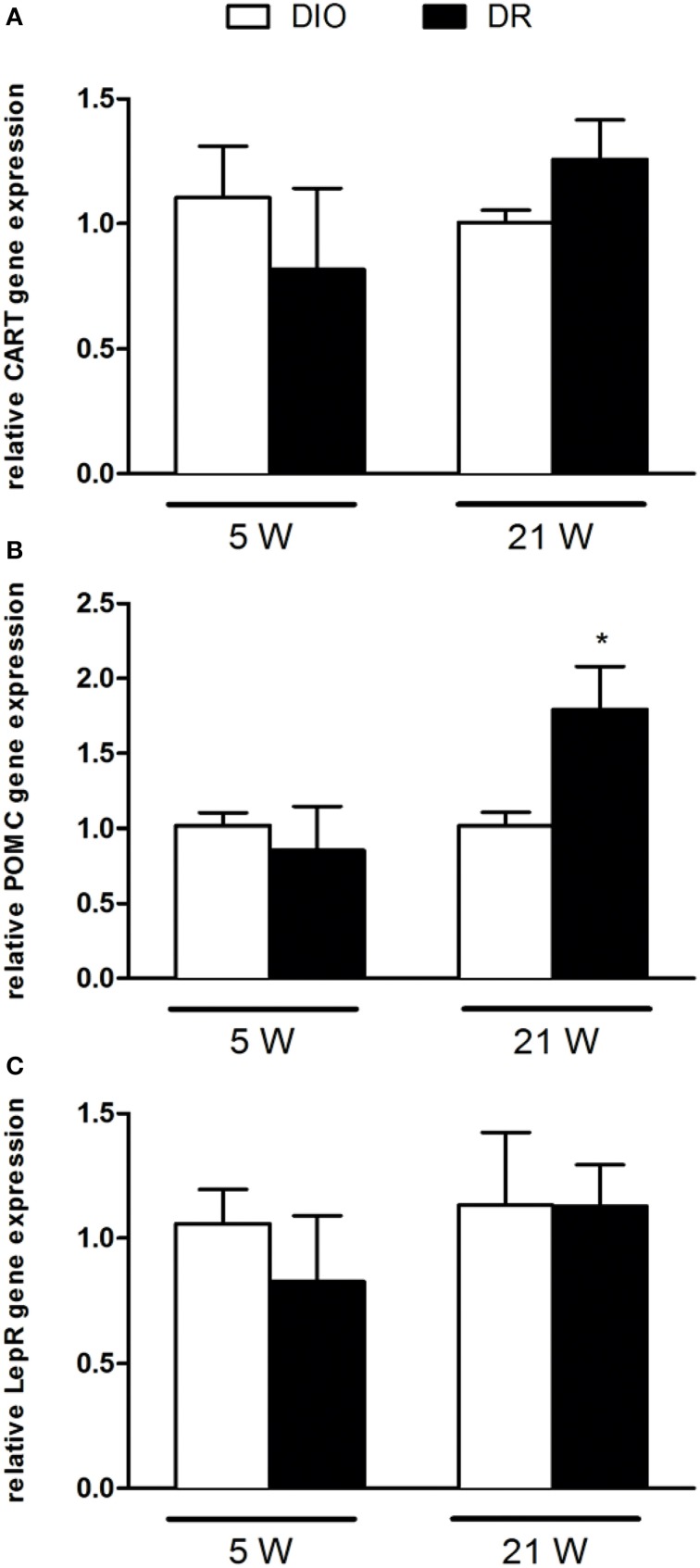
**RT-qPCR analysis of (A) CART, (B) POMC, and (C) LepR gene expression after 5 and 21 weeks of high fat diet exposure**. Gene expression was normalized to glyceraldehyde-3-phosphate dehydrogenase and β-actin. Values are expressed as mean ± SEM of 6–8 rats. ^*^P < 0.05 vs. DIO.

After 5 and 21 continuous weeks of free access to high fat diet, we detected the gene expression of NPY, AGRP, POMC, CART peptide precursors, as well as of the two receptors PPAR-γ and lepR (Figures 2, 3).

As reported in Figure 2, there was a significant decrease of NPY [*F*_(1, 9)_ = 9.45; *P* < 0.05] and PPAR-γ [*F*_(1, 9)_ = 8.78; *P* < 0.05] mRNA levels in the hypothalamus of DR rats respect to DIO rats sacrificed after 5 weeks of high fat diet exposure (Figures 2A,C). No changes were detected in AGRP gene expression levels [*F*_(1, 9)_ = 0.52; *P* > 0.05] (Figure 2B). In Figure 3, we reported the gene expression analysis of POMC, CART, and lepR that was not different between DIO and DR rats at 5 weeks (Figure 3A). After 21 weeks of free access to high fat food, a significant increase was observed only in POMC gene expression in DR rats [*F*_(1, 9)_ = 5.54; *P* < 0.05] respect to DIO group (Figure 3B).

### DNA methylation at gene promoters

In order to evaluate the potential relationship between the observed significant alterations in genes expression and epigenetic regulation, we analyzed the DNA methylation at gene promoters of NPY, PPAR-γ (5 weeks) and POMC (21 weeks).

**Figure 4:**
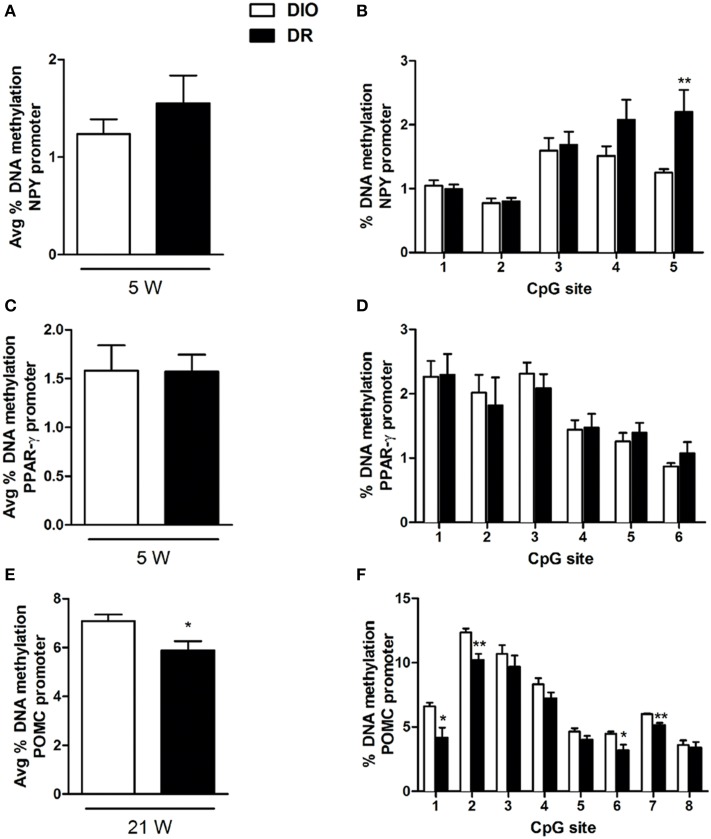
Percentage of DNA methylation assessed with bisulfite pyrosequencing in the hypothalamus of rats for: NPY gene promoter at all CpGs combined (A) and at each single site (B) (^**^P < 0.01 vs. DIO) after 5 weeks of high fat diet exposure; PPAR-γ gene promoter at all CpGs combined (C) and at each single site (D) after 5 weeks of high fat diet exposure; POMC gene promoter at all CpGs combined (E) and at each single site (F) (^*^P < 0.05, ^**^P < 0.01 vs. DIO) after 21 weeks of high fat diet exposure. Values are expressed as mean ± SEM of 6–8 rats.

Methylation of the combined CpG sites examined in the promoter region of NPY at 5 week (from −3 to −36 upstream the transcription start site) did not show significant difference in the % of DNA methylation in DR rats respect to DIO groups [*F*_(1, 8)_ = 0.98; *P* > 0.05] (Figure 4A). The overall ANOVA of 5 CpG sites present on NPY promoter region showed significant changes between the experimental groups (Groups: [*F*_(1, 8)_ = 9.91; *P* < 0.05]; CpG [*F*_(4, 32)_ = 14.41; *P* < 0.01]; Interaction: [*F*_(4, 32)_ = 3.15; *P* < 0.05]). *Post-hoc* analysis revealed a selective significant increase in DNA methylation for the 5th CpG site in the DR rats (as shown in Figure 4B).

Methylation of the combined CpG sites examined in the promoter region of PPAR-γ at 5 week (from −387 to −434) did not show significant difference in the % of DNA methylation in DR rats respect to DIO groups [*F*_(1, 8)_ = 0.001; *P* > 0.05] (Figure 4C). The overall ANOVA of 6 CpG sites present on PPAR-γ promoter region did not show significant changes between the experimental groups (Groups: [*F*_(1, 10)_ = 0.001; *P* > 0.05]; CpG [*F*_(5, 50)_ = 11.44; *P* < 0.05]; Interaction: [*F*_(5, 50)_ = 0.32; *P* > 0.05]) (Figure 4D).

Methylation of the combined CpG sites examined in the promoter region of POMC at 21 week (from −1 to −59) showed significant reduction in the % of DNA methylation in DR rats respect to DIO groups [*F*_(1, 6)_ = 6.85; *P* < 0.05] (Figure 4E). The overall ANOVA of 8 CpG sites present on POMC promoter region showed significant changes between the experimental groups (Groups: [*F*_(1, 6)_ = 6.85; *P* < 0.05]; CpG [*F*_(7, 42)_ = 142.1; *P* < 0.01]; Interaction: [*F*_(7, 42)_ = 2.32; *P* < 0.05]) (Figure 4E). *Post-hoc* analysis revealed a selective significant decrease in DNA methylation levels at four specific CpG sites, respectively in the 1st and 6th, and in the 2nd and 7th CpG sites (Figure 4F).

## Discussion

Obesity results from the complex interaction of genetic components and environment, which facilitates the development of obese phenotype (Perusse and Bouchard, 2000). High-fat diet is among the most important environment factors leading to obesity and models of DIO are commonly used to study the disease. Our findings confirmed that the outbred Sprague Dawley rats exhibited different phenotype after exposed to high-fat diet and the body weight resulted significantly increased in DIO rats when compared to DR rats starting after 5 weeks of exposure and up to 21 weeks. Previous studies indicated that increased energy intake should be the primarily responsible for body weight gain in DIO rats on high-fat diet *ad libitum*, whereas DR rats exposed to high-fat diet compensated for the increased energy density of the high-fat diet by eating significantly less (Levin et al., 1989; Ricci and Levin, 2003). In our study, food intake of DR rats showed a clear trend to be lower than food intake of DIO rats but the difference was statistically significant only at one time point (4th week).

To evaluate the possible mechanisms responsible of obesity development, we investigated the expression of selected hypothalamic neuropeptides and receptors known to be involved in in the control of body weight.

In the complex system of hypothalamic signals regulating energy homeostasis, we evaluated if there is also an interconnection in gene regulation between NPY/AgRP and POMC/CART, where the NPY/AgRP neurons might down-regulate POMC neurons (Cowley et al., 2001).

Among the different genes under investigation, we observed, after 5 weeks high fat diet exposure, a selective down-regulation of NPY and PPAR-γ genes expression in DR when compared to DIO rats, and no changes for AgRP as well as for all the anorexigenic genes mRNA levels.

NPY is one the most potent orexigenic peptides in the brain and, consistently with our findings, previously it has been observed that high-fat-sucrose diet in prenatally stressed female adult rats induces an increase in NPY mRNA levels (Paternain et al., 2012). Moreover NPY mRNA levels were found to be higher also in DIO group when compared to DR group in the hypothalamus (Wang et al., 2007). Interestingly, NPY knockdown in the hypothalamus increases energy expenditure (Chao et al., 2011) and its overexpression within the paraventricular nucleus induced obesity via increased food intake (Tiesjema et al., 2009).

Instead, we did not observe alterations in AgRP gene expression which it is synthesized exclusively in the arcuate nucleus (Morton and Schwartz, 2001; Valassi et al., 2008), where it co-localizes with NPY.

In this work we studied the entire hypothalamic region and NPY is also expressed in other hypothalamic areas apart from the arcuate and this could be the reason why we observed changes only in this gene.

Consistently with NPY gene expression changes, we report a reduction in PPAR-γ mRNA levels in DR. Again, this finding is in agreement with a previous study showing that hypothalamic PPAR-γ gene expression is higher in DIO mice when compared with lean controls (Diano et al., 2011). The deletion of PPAR-γ gene in neurons (Lu et al., 2011) or its chemical inhibition in the hypothalamus, protects against the development of DIO (Ryan et al., 2011). Moreover, during high-fat diet feeding, food intake was reduced and energy expenditure increased in PPAR-γ KO mice resulting in reduced weight gain (Lu et al., 2011).

Interestingly, we have observed that these alterations are time-dependent since these were not evident anymore at the latest time-point under study (21 weeks), but just when the obese phenotype starts to develop. It could be hypothesized that NPY and PPAR-γ orexigenic role is not any longer of relevance once the obese phenotype is established. This observation might be even more relevant when taking into account the possible epigenetic regulation of these genes expression. In fact, even if there is globally a hypomethylation in all animals consistent with the observations of others (Plagemann et al., 2009; Mahmood et al., 2013), we found an even lower DNA methylation at NPY gene promoter in DR animals. Changes in DNA methylation of specific CpG sites at NPY gene promoter in the hypothalamus of rats on a high-carbohydrate milk formula have also been recently reported (Mahmood et al., 2013). Instead, no changes in NPY gene expression, as well as promoter DNA methylation, was observed in a rat model for the study of the effects of overnourishment during the suckling period (Plagemann et al., 2009). In addition, others reported altered expression of hypothalamic neuropeptide genes such as Npy, Agrp, and Pomc but non-correlated with DNA methylation changes (Shin et al., 2012).

One important point is that changes in gene expression regulation are selective, since no alterations have been observed for the other genes under study, and selectively limited at the 5 weeks time-point. We might thus hypothesize that orexigenic genes (NPY and PPAR-γ in this study), if activated in early events, would be responsible of the maintenance of the obese phenotype even if their regulation is not needed anymore when this phenotype is already well-established. Moreover, the epigenetic regulation of NPY might suggest that the overexpression is reversible via environmental stimuli and thus obesity development could be avoided if early counteracted.

After 21 weeks exposure to high fat diet, we observed that the expression of most of the genes under study resulted not be different between DIO and DR, beside the lower mRNA levels of the anorexigenic POMC gene in DIO rats when compared to DR. A reduction in POMC gene expression was also observed in the hypothalamus of female rats on high-carbohydrate milk formula (Srinivasan et al., 2008; Mahmood et al., 2013). It is known that α-MSH, derived from POMC, inhibits food intake and enhances energy expenditure mainly through activation of melanocortin receptors in the hypothalamus and that it is effective especially against adult-onset obesity (Hansen et al., 2001; Hwa et al., 2001; Zhang et al., 2004). This is of relevance in the frame of our findings of POMC gene regulation at the longest time-point to demonstrate its importance on long-term body weight maintenance.

Moreover, we here show that POMC expression at week 21 is strongly influenced by promoter methylation, as already reported by others (Newell-Price et al., 2001). In fact, we observed, in DR group when compared to DIO rats, significant reductions of DNA methylation at four of the eight CpG sites investigated in the proximal promoter region of POMC, thus negatively correlated with the observed increase in gene expression. This is in agreement with other studies, using different animal models. Namely, in an animal model of overnutrition during the suckling period, a CpG dinucleotide located in the GRE-binding site, a negative regulator of Pomc expression, resulted hypomethylated (Plagemann et al., 2009). In another rodent model, feeding of a low-protein diet to pregnant rats induced a mismatched correlation between POMC gene expression and CpG methylation status in the offspring (Coupe et al., 2010). However, others also reported that in high-fat diet-fed obese mice, altered expression of POMC and LepR in the hypothalamus was not to correlated with changes in DNA methylation at gene promoters (Fan et al., 2011).

## Conclusions

We here provide evidence of selective and time dependent transcriptional regulation of target genes in DIO rat model when compared to DR. These alterations in peptide genes regulation would contribute to develop overweight in rats possibly for the hedonic impact of palatable food in DIO rats when compared to DR. It is important to underline that observed changes appear to be relevant at the earliest time-point under study. This allows to hypothesize that it is crucial to identify alterations in gene transcription at the very beginning of obesity development, if one aims at predicting disease trajectories and choosing the most effective therapy. Moreover, the reversible nature of epigenetic modifications makes them attractive targets for a possible epigenetic therapy of obesity. In addition, understanding how environmental factors, as in this animal model, might induce obesity would be of help to disclosure changes occurring in central circuits. Such an epigenetic regulation appears to hold better promises than alterations detected in genetic models of obesity (Kalra et al., 1999), which most often do not reproduce the changes occurring in natural populations affected by the disease.

Further studies are needed to dissect gene expression regulation and DNA methylation patterns of other possible genes involved in obesity development, possibly looking at distinct target tissues, not only within the CNS, but also at the periphery (adipose tissue, liver etc.).

### Conflict of interest statement

The Editor Luca Steardo declares that, despite being affiliated to the same institution as author Adele Romano, the review process was handled objectively and no conflict of interest exists.
