# Supplementary material for: Regulation of hypothalamic neuropeptides gene expression in diet induced obesity resistant rats: possible targets for obesity prediction?
Source: Front Neurosci. 2015 Jun 8;9:187. doi: 10.3389/fnins.2015.00187 (PMC4458694; doi:10.3389/fnins.2015.00187)
Supplement: Supplementary file 1 [file Table1.DOCX]

**Table 1S**

| **Gene** | **Primers (5’ – 3’)** |  |
| --- | --- | --- |
| **β-ACT** | Fwd: AGATCAAGATCATTGCTCCTCCT  Rev: ACGCAGCTCAGTAACAGTCC | Gene expression |
| **GAPDH** | Fwd: AGACAGCCGCATCTTCTTGT  Rev: CTTGCCGTGGGTAGAGTCAT | Gene expression |
| **NPY** | Fwd: CCGCCCGCCATGATGCTAGG  Rev: GGCCATGTCCTCTGCTGGCG | Gene expression |
|  | Rn_Npy_02_PM PyroMark CpG assay (PM00550025) | DNA Methylation |
| **AGPR** | Fwd: GTTTCCAGGAACCCAAGGG  Rev: GCATGGCCTTTGCTTGTT | Gene expression |
| **PPAR-γ** | Fwd: GCTGGCCTCCCTGATGAATA  Rev: GCTTCCGCAGGCTTTTGA | Gene expression |
|  | Rn_Pparg_03_PM PyroMark CpG assay (PM00549535) | DNA Methylation |
| **CART** | Fwd: GGACATCTACTCTGCCGTGG  Rev: GCGTCACACATGGGGACTT | Gene expression |
| **POMC** | Fwd: GACCAAACGGGAGGCGACGG  Rev: GGCTCTGTCGCGGAAAGGCA | Gene expression |
|  | Fwd: GGTTGGGTGGGTGAGTTT  Rev (Biotin): CCTCCCCACTTTCCAAACACATCTACTAT  Seq: GGTGGGTGAGTTTTG | DNA Methylation |
| **LepR** | Fwd: CCAGTACCCAGAGCCAAAGT  Rev: GGGCTTCACAACAAGCATGG | Gene expression |
